# ERCC1表达对Ⅰ期非小细胞肺癌患者术后生存率的影响

**DOI:** 10.3779/j.issn.1009-3419.2010.05.26

**Published:** 2010-05-20

**Authors:** 征平 丁, 杰 张, 晋晨 邵

**Affiliations:** 1 200030 上海，上海市胸科医院/上海市肺部肿瘤临床医学中心胸外科 Department of Toracic Surgery, Shanghai Chest Hospital/Shanghai Lung Tumor Clinical Medical Center, Shanghai 200030, China; 2 200030 上海，上海市胸科医院 Department of Pathology, Shanghai Chest Hospital/Shanghai Lung Tumor Clinical Medical Center, Shanghai 200030, China

**Keywords:** 肺肿瘤, 核苷酸切除修复交叉互补, 预后, Lung neoplasms, Excision repair cross-complementing, Prognosis

## Abstract

**背景与目的:**

核苷酸切除修复交叉互补（excision repair cross-complementating, *ERCC*）基因家族能对核苷酸进行切除和修复以减少DNA的损伤。如果损伤性的核苷酸切除修复机制受损，可增加基因组的不稳定性，进而导致肿瘤发生更多的恶性表型行为。因此本研究旨在评价肿瘤ERCC1的表达对早期非小细胞肺癌（non-small cell lung cancer, NSCLC）手术切除后患者的生存率的影响。

**方法:**

采用免疫组化方法检测ERCC1在肿瘤中的表达，以评估ERCC1表达对118例接受过根治性切除的Ⅰ期NSCLC患者生存率的意义。

**结果:**

肿瘤ERCC1表达阳性与阴性的患者3年生存率分别为86.12%、65.46%，两者差异具有统计学意义（*P*=0.025）。多变量分析表明，ERCC1高表达对较长生存期具有独立预测性。

**结论:**

手术切除的NSCLC患者中，ERCC1阳性表达者的生存率较阴性表达者高。可见，完整的DNA修复机制可减少导致肿瘤恶性潜能的基因畸变的积累，从而减少复发的危险。

## Abstract (en)

**Background and objective:**

Proteins of the nucleotide excision repair pathway can repair DNA damage. The excision repair cross-complementing (*ERCC*) gene family reduce damagement of DNA by nucleotide excision and repair. The aim of this study is to investigate the expressions of ERCC1 (members of DNA repair gene family) in patients with non-small cell lung cancer (NSCLC) as well as their clinical prognostic significance.

**Methods:**

Expression levels of ERCC1 were detected by IHC in 118 stage Ⅰ NSCLC patients. *Kaplan-Meier* survival curve, and *Cox* multivariate regression analysis were used for statistical analysis.

**Results:**

The patients with high expression of ERCC1 had significantly longer survival time than those with low expression of ERCC1, and *Cox* multivariate regression analysis showed that expression of RRM1 was an independent prognostic factor for NSCLC patients.

**Conclusion:**

NSCLC patients with high ERCC1 expression have a better survival when compared to patients with low ERCC1 expression. Therefore, an intact DNA repair mechanism may reduce the accumulation of genetic aberrations that are thought to contribute to a tumor malignant potential and therefore the risk of relapse after definitive treatment.

肺癌是目前人类癌症死亡的最主要原因之一。全世界每年有500万人死于肺癌。近20年来，中国肺癌的发生率也在明显上升。肺癌中约80%为非小细胞肺癌（nonsmall cell lung cancer, NSCLC）。早期NSCLC患者完全手术切除后，5年生存率大约为30%-70%，存在很大的异质性，这引起了人们的思考。肺癌的临床期别仍是最重要的临床预后指标之一，同时也是极为重要的治疗预测因子，但对于每一个体而言，这种预后标记并不严密。当前很多研究着重于寻找与疾病复发相关的分子标记，以便筛查出潜在获益的人群进行更加个体化的治疗。此外，要进一步提高完全切除术后的中早期NSCLC患者的长期生存率，尚需要寻找新的可能的靶点并发展新的治疗手段。

对NSCLC生物学特性更多的了解，有助于预测肿瘤的复发以及选择对复发、生存率和生活质量效果最佳的治疗性干预措施。核苷酸切除修复途径中的蛋白质可以修复铂类药物引起的DNA损伤。核苷酸切除修复交叉互补（excision repair cross-complementing, *ERCC*）基因家族通过核苷酸进行切除和修复以减少DNA的损伤。其过程首先是将修饰过的核苷酸连同毗邻的核苷酸一起从损伤的核苷酸链移除，随后在DNA聚合酶的作用下合成完好如初的核苷酸链^[1]^。*ERCC1*基因编码一种含有297个氨基酸的蛋白质，这种蛋白质通过与XPF和ERCC4共同组成一复合体而发挥作用^[2]^。该复合体可能是重组性修复和核苷酸切除修复过程所必需的^[3, 4]^。核切除修复功能受损可增加基因组的不稳定性，进而导致肿瘤发生更多的恶性表型行为。癌症基因的不稳定性主要表现为癌症患者的染色体结构畸变（即缺失、转位和插入）。当DNA双链断口通过同源性重组和非同源性末端连接方式进行损伤性修复时即可出现上述染色体结构畸变现象。本研究采用免疫组化方法检测ERCC1在肿瘤中的表达，以评估ERCC1表达对118例接受过根治性切除的I期NSCLC患者生存率的意义。

## 1 材料与方法

### 1.1 临床与病理资料

对118例2007年1月-2009年1月期间就诊于上海市胸科医院肺部肿瘤临床医学中心手术的Ⅰ期NSCLC患者进行回顾分析，排除病理类型不详的患者（表 1）。术前分期工作包括：血液生化检查、胸部CT扫描、上腹部B超和CT、骨同位素扫描、纤维支气管镜检查、头颅CT或MRI检查。疑似有远处转移的患者均被排除。所有患者均接受完整手术切除，支气管切断均为阴性。

**1:** 患者的一般特征 Patient Characteristics

Characteristics	*n*
Gender	
Male	65
Female	53
Age	
Median age	65
Pathological type	
Squamous	39
Adenocarcinoma	59
Squamous adenocarcinoma	20
Smoking status	
Non-smoking	48
Smoking	70
ERCC1	
Negative	38
Positive	80

### 1.2 免疫组化

免疫组化染色采用Envision法。挑选相应存档蜡块，连续石蜡切片厚4 μm。按EnVision法进行免疫组织化学法检测。石蜡切片常规脱蜡水化，pH6.0柠檬酸加热抗原修复；3%H_2_O_2_阻断过氧化物酶；加入一抗50 μL/片，4 ℃过夜，ERCC1鼠抗1:80TBS稀释（ERCC1购自Abcom公司）；加入二抗50 μL/片，室温60 min；DAB显色5 min-10 min。各步骤间均用TBS缓冲液冲洗5 min×3次，苏木素衬染，烤干、封片。ERCC1阳性定位于细胞核；阳性细胞 < 10%记为“-”，阳性细胞 > 10%记为“+”。以已知阳性切片作为阳性对照，以TBS缓冲液代替一抗作为阴性对照。

### 1.3 统计学分析

数据以SPSS 11.0统计软件进行统计处理，用*Kaplan-Meier*进行生存分析，*Log-rank*检验分析生存差异，建立*Cox*回归分析了解患者的预后相关因素。卡方检验、*Fisher’s*确切概率法进行显著性检验，*Spearman*等级相关进行相关性分析。*P* < 0.05为差异有统计学意义。

## 2 结果

### 2.1 免疫组化结果

如图 1所示。

**1:**
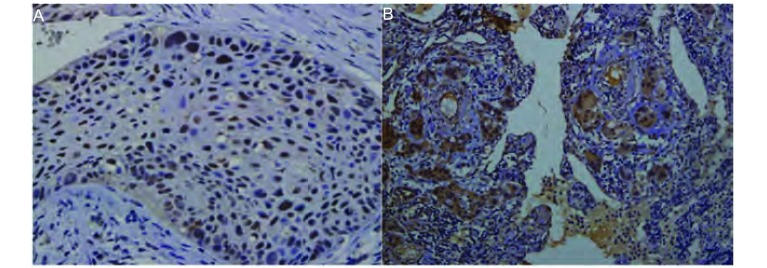
ERCC1免疫组化结果。A：ERCC1阳性患者的染色（×400）；B：ERCC1阳性患者的染色（× 100） Immunohistochemical results of ERCC1. A: ERCC1 positive expression in NSCLC tissue (× 400); B: ERCC1 positive expression in NSCLC tissue (×100)

### 2.2 临床随访

最终随访日期为2010年3月20日，中位随访时间为34.14个月，118例患者的中位年龄为65岁，未有患者接受术后化疗。生存期定义为患者手术之日至由于任何原因死亡或是末次随访时间。

### 2.3 总体生存期（overall survival, OS）

ERCC1阳性的患者（*n*=80）3年生存率优于ERCC1阴性的患者（*n*=38）（86.12% *vs* 65.46%; *P*=0.025）（图 2）。*Cox*多因素分析提示年龄、性别、吸烟史、ERCC1表达情况是影响患者预后的因素（表 2）。

**2:**
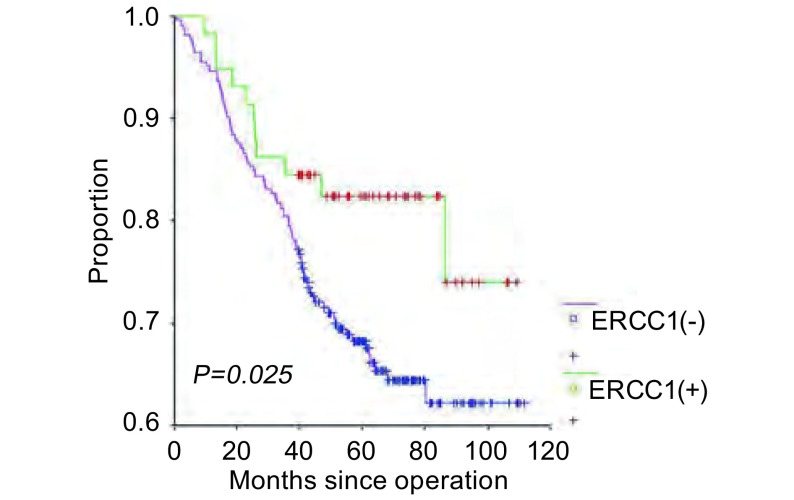
不同ERCC1水平的患者生存情况 Patients survival according to ERCC1 status

**2:** 影响患者生存状况的多因素分析 Multivariate analysis of prognostic factors

	HR (95%CI)	*P*
Age^*^		0.012
< 65		
> 65	2.129 (1.371-3.222)	
Gender		0.043
Female		
Male	2.225 (1.180-3.969)	
Pathological type		0.250
Squamous		
Adenocarcinoma	0.646 (0.354-1.107)	
Squamous adenocarcinoma	2.010 (0.268-18.200)	
Smoking history		0.025
No		
Yes	3.468 (0.799-2.549)	
ERCC1		0.023
Negative		
Positive	2.068 (1.107-3.864)	
^*^: Median age.		

## 3 讨论

本研究中评价了肿瘤内ERCC1表达对NSCLC患者经手术切除后生存期的影响。ERCC1属于核切除修复基因家族，它编码一种蛋白质，这种蛋白质能与修复复合体的其他它成员协同通过修复核苷酸的化学变化和结构改变以确保基因组的完整性。我们发现，对于经手术切除的NSCLC患者来说，ERCC1表达的增加是生存率改善的一个显著且独立的预测指标。我们认为，正是肿瘤内的ERCC1参与了肿瘤DNA的修复，从而影响了肿瘤的生物学行为。

关于ERCC1表达影响肿瘤生物学行为的机制，我们归之为ERCC1修复细胞内DNA损伤的能力。当前关于肿瘤发生和发展的学说是假定基因损伤不断地在上皮细胞内累积。已发表的文献报道^[5, 6]^也表明，伴有DNA倍体异常、微卫星不稳定性和等位基因缺失的多种基因组变化使肺癌更多出现生长速度加快和远处转移等恶性表现。肺癌的基因组改变纷繁复杂，从数量极少但生物学意义极为重要的基因畸变到广泛的基因组损伤皆可发生。该损伤的程度可能取决于接触致癌物的类型和剂量以及细胞自身修复该损伤的能力。与那些损伤较小的细胞相比，发生广泛损伤的细胞，逃避了机体正常的趋凋亡监控机制，具有增殖优势和更多恶性表型行为。本研究中所测定的DNA损伤修复基因*ERCC1*，可能对细胞自身的DNA损伤修复能力具有代表性，因而也是肿瘤内累积的DNA损伤程度的间接性生物标记物。

然而，ERCC1表达的增加也预示着对顺铂抗药性的增加，这是与ERCC1在损伤核苷酸修复中的作用相吻合的，尤其是ERCC1能使顺铂诱导的DNA络合物的清除增加^[7, 8]^。因此，对于那些接受过以顺铂为基础的化疗的进展期癌症患者，ERCC1表达增加的结果是促进铂类诱导的DNA络合物的清除，从而降低了疗效和生存率。

临床研究表明，含铂方案辅助化疗可使完全切除的非小细胞肺癌患者生存受益。完全切除术后的非小细胞肺癌术后含铂方案辅助化疗可改善5年生存，延长5年生存率4%-15%。但是临床研究^[9, 10]^也显示，术后含铂方案辅助化疗有较多的不良反应，不同患者对治疗的耐受性也有很大差别，且术后辅助化疗的结果有很强的异质性，不同分期和肿瘤负荷的患者术后辅助化疗结果有很大的差别，只有部分患者可从中获益。

总之，ERCC1在肺癌的发生发展及临床治疗方面的作用正逐渐清晰，ERCC1表达的水平对于生存状况具有预测意义。这可能是由于其对DNA损伤卓有成效的修复作用减少了基因组损伤累积的结果。但是，ERCC1水平的增加同时也降低了以铂类为基础的化疗方案的益处，一旦被证实，那么将来关于辅助治疗和新辅助化疗的试验设计均需考虑到上述结论。由于低ERCC1表达患者生存率相对较低，但肿瘤对铂类为基础的化疗方案相对敏感，因此这部分患者将可能从铂类为基础的化疗方案中获得最大收益。
